# Strategies for Nitrite Replacement in Fermented Sausages and Effect of High Pressure Processing against *Salmonella* spp. and *Listeria innocua*

**DOI:** 10.3390/foods10112617

**Published:** 2021-10-28

**Authors:** Constanza Maria Lopez, Giuliano Dallolio, Paolo Bonilauri, Annalisa Rebecchi

**Affiliations:** 1 Department for Sustainable Food Process, Università Cattolica del Sacro Cuore, Via Bissolati 72/74, 26100 Cremona, Italy; constanzamaria.lopez@unicatt.it (C.M.L.); giuliano.dallolio@gmail.com (G.D.); 2 Istituto Zooprofilattico Sperimentale della Lombardia ed Emilia-Romagna (IZSLER), Via Bianchi 9, 25124 Brescia, Italy; paolo.bonilauri@izsler.it

**Keywords:** nitrite-free fermented sausages, High Pressure Processing (HPP), *Listeria innocua*, *Salmonella* spp.

## Abstract

The development of nitrite-free meat products is a current industrial concern. Many efforts have been attempted to replace the nitrite effect in cured meats colour formation and pathogens control. Our previous work evidenced that lactic acid and a cold ripening were the best hurdle technologies for nitrite-free fermented sausages from metabolomics. In the first part of this work, we investigated the effect of lactic acid compared with both two alternative additives (glucono-D-lactone and a mix of sodium di-acetate/sodium lactate) and with low-nitrite sausages, all of them following either cold or traditional ripening. For this purpose, microbiological analysis, pH, water activity (a_w_), and a sensory study were performed. All nitrite-free sausages (cold or traditional ripened) showed quality and safety traits similar to low-nitrite traditionally ripened ones used as control. In addition, sensory study revealed that sausages with lactic acid were the most preferred cold ripened samples, supporting that this is an optimal strategy for the production of nitrite-free sausages. We selected this product for further studies. Indeed, in the second part, we evaluated the impact of ripening, and other hurdle technologies as High Pressure Processing (HPP) and under-vacuum storage against *Listeria innocua* and *Salmonella* spp. by a challenge test. Maximal declines were obtained for ripening along with HPP (i.e., 4.74 and 3.83 log CFU/g for *L. innocua* and *Salmonella* spp., respectively), suggesting that HPP might guarantee nitrite-free sausages safety. Although the quality of raw materials remains essential, these hurdle strategies largely contributed to nitrite-free sausages safety, offering a promising tool for the meat industry.

## 1. Introduction

Nitrites and nitrates have long been used to avoid spoilage and to extend shelf life of meat products. As curing agents, they contribute to meat products colour and flavour. Nitrites have played a key role in inhibiting *Clostridium botulinum* spores and in preventing food poisoning caused by their toxin [1,2]. Recent findings suggest that nitrites could have an antagonistic effect against *Listeria* and *Salmonella*, two major pathogens in meat products, often used as microbiological safety standards [3]. In particular, nitrites and nitrates led to the inhibition of *L. innocua* in dry cured ham [4], as well as *L. monocytogenes* and *S. enterica* serovar Thyphimurium in dry fermented sausages [5,6]. Apart from their multiple benefits, nitrites might produce carcinogenic and mutagenic compounds, such as N-nitrosamines, after a series of chemical reactions in the meat matrix [7]. The concentration of these undesirable compounds greatly depends on the quantity of nitrites and nitrates added to the meat batter, but it is also affected by raw materials quality, processing, and other factors [8]. In this sense, the European Union allows maximal concentrations of 250 ppm of NaNO_3_, or 150/150 ppm of NaNO_3_/NaNO_2_ for traditional products, as established in EC 1129/2011 [9]. On the other hand, meat products labelled as organic foods can have a maximal concentration of 80 ppm of nitrites in the European Union according to EC 780/2006 [10], while in the U.S., the addition of nitrites and nitrates in organic products is forbidden [11].

In the last few years, driven by consumer demands for healthier foods, there has been an increased interest in the development of low-nitrite or nitrite-free meat products, but consumer safety and preserving meat products typical organoleptic characteristics have to be guaranteed. Therefore, a strict selection of raw materials becomes essential to accomplish high-quality standards in nitrite-free products. Since the production chain usually implies a natural contamination, the combination of synergistic hurdle technologies that inactivate or decrease foodborne pathogens without affecting nutritional or sensory traits is required [12,13]. In practice, this means improving the manufacturing process by adjusting diverse variables, for example: (i) temperature and time of fermentation and ripening, (ii) final water activity (a_w_) and pH values, (iii) use of selected starter culture, and (iv) addition of safe antimicrobial agents such as ascorbic or lactic acids, sodium lactate, sodium acetate, etc.

In addition, to validate the ready-to-eat (RTE) meat-derived food process for export to the U.S. that requires a reduction of 5-log CFU/g [14], additional treatments could be necessary. In this regard, High Pressure Processing (HPP) is currently becoming an industrial reality as an additional hurdle for fermented sausages preservation [15,16,17]. In fact, HPP represents the only non-thermal process with commercial impact, even when major advances in these technologies have been made around the world [18]. In general, HPP applies a pressure range from 100 to 1000 MPa transmitted by a liquid to a packaged product that inactivates bacteria but only partially inactivates spores. Several mechanisms are implied, such as protein denaturation, although they are not completely understood so far [19,20,21]. Many authors proposed that this process is able to reduce pathogen bacteria while both technological microbiota and typical sensory characteristics of fermented sausages remain unaltered [22,23].

Actually, there is some evidence that HPP can significantly inhibit *Listeria* sp. and *Salmonella* spp. [16,24,25,26,27]. Thus, the replacement of nitrites antagonistic effect by this technology may be feasible. Meanwhile, Balamurugan et al. [28] warned about some intrinsic aspects of fermented sausages that may decline the efficiency of HPP—for instance, the complexity of the food product, the high concentrations of NaCl, and the low a_w_ values. In addition, resistance to HPP seems to be a strain-dependent characteristic [15,29]. Considering all these facts, the performance of HPP treatment should be individually evaluated for each product by a challenge test to provide insights about the control of undesirable bacteria.

Recently, our group evaluated a novel cold ripening process and the addition of both ascorbic and lactic acids as hurdle technologies in nitrate-free salami [30]. We used a metabolomic approach to find those conditions that avoided case hardening and extreme oxidation of samples. In addition, we hypothesised that this improved process represented an interesting alternative to nitrate/nitrite addition, even though further studies were recommended, such as safety assessment or additional hurdle technologies for final products.

The aim of this study was to evaluate lactic acid addition for nitrite-free sausage production in comparison to two uncommon additives (glucono-D-lactone and a mix of sodium di-acetate/sodium lactate), and low-nitrite sausages, all of them following either cold or traditional ripening. The best strategy for nitrite replacement in fermented sausages was selected for further studies. We investigated the contribution of ripening and other hurdle technologies as under-vacuum storage, and HPP in *Listeria innocua* and *Salmonella* spp. inactivation by a challenge test.

## 2. Materials and Methods

### 2.1. Manufacturing of Fermented Sausages with Different Additives

Fermented sausages were produced in triplicate in a small-scale manufacturer in Northern Italy (Salumificio Santini, Cremona, Italy). The meat came from “Gran Suino Padano PDO”, a typical Italian heavy pig with an age of over 9 months and a live weight of over 150 kg, well suited to the production of Italian fermented meat sausages. A mixture of 100 kg was prepared according to Rocchetti et al. [30]: pork meat (pork leg, defatted boneless pork shoulder, pork neck, 73%) and fatty tissues (pork skinned belly, pork throat fat, 23.7%) trimmed 6 mm, salt (2.5%), white wine (0.3%), ascorbic acid (0.2%), dextrose (0.15%), black pepper (0.08%), and garlic (0.01%). Starter cultures (Teracell, Cremona, Italy) were added in a final concentration of 1 × 10^7^ CFU/g for *Lactobacillus sakei* and 3 × 10^6^ CFU/g for two species of staphylococci coagulase negative (CNS), *Staphylococcus xylosus* and *Staphylococcus carnosus*. The total meat batter was then divided into five batches (20 kg each), each one with different additives: (1) lactic acid 0.3% (Chimab, Padova, Italy), (2) glucono-D-lactone 0.6% (Chimab, Padova, Italy), (3) a mix of sodium di-acetate (E 262) and sodium lactate (E 325) 1.5% (Opti.Form powder 98, Corbion, The Netherlands), (4) vegetable extracts containing nitrites 80 ppm (Accel^TM^, Kerry, Ireland), and (5) sodium nitrite 80 ppm (Chimab, Padova, Italy). These last two batches were prepared according to the maximal concentration allowed for organic fermented sausages in Europe and used as low-nitrite controls.

Then, the mixtures were stuffed into 38 mm diameter collagen casings using a vacuum filler (Handtmann, Biberach, Germany), resulting in sausages of 20–22 cm length that weighed 370 g, approximately. All the batches (from 1 to 5) were divided into two groups that followed two different ripening processes in drying cabinets (Everlasting, Mantova, Italy) for 35 days, for a total of 10 baches. The “traditional ripening” group (T) followed a first phase set at decreasing temperatures from 22–14 °C and 65–90% relative humidity (RH) for 3–5 days and a second step at 14–12 °C and 75–85% RH for 30–32 days. The “cold ripening” (C) group followed a first step with a temperature range of 6–8 °C and 65–90% RH for 20–25 days, until the a_w_ value reached ≤0.92, while the second phase had a temperature range of 10–12 °C and 75–85% RH for 10–12 days. This last process was designed in our previous study [30] to avoid the growth of *Clostridium botulinum* belonging to Group I or II, as previous studies had found [31]. On the other hand, the traditional ripening provided the standard parameters to evaluate the suitability of the studied cold ripening. Samples were collected before stuffing at time 0 (t_0_) and after 6 days (t_6_), 15 days (t_15_), and 35 days (t_35_). Samples of each formulation/condition from the three replicates at a defined time point were taken, weighed, and analysed. For each sample, three sub-samples (from central and at both ends) were pooled and homogenised, then an aliquot was used for further analyses.

### 2.2. Microbiological Analysis, pH, and Water Activity

For microbial enumeration, 10 g of fermented sausage samples were aseptically removed and diluted 1:10 with saline water (0.9% NaCl) and homogenised for 1.5 min at 260 rpm in a Stomacher Lab-Blender (400 Circulator; International PBI, Milan, Italy). Briefly, appropriate decimal dilutions were plated in duplicate onto the following media (Oxoid, Milan, Italy) and incubated under these conditions: Violet Red Bile Glucose Agar (VRBGA) for 24 h at 37 °C for *Enterobacteriaceae* (ISO 21528-2, 2017) [32], Violet Red Bile Agar (VRBA) for 24 h at 37 °C for total coliforms, VRBA supplemented with MUG 100 µg/mL for 24 h at 44 °C for *Escherichia coli*, MRS Agar for 72 h at 30 °C under restricted oxygen conditions achieved using Anaerocult A (Merck, Darmstadt, Germany) for lactobacilli (ISO 15214, 1998) [33] and Baird Parker Agar added with egg yolk tellurite emulsion at 37 °C for 48 h in aerobic conditions for staphylococci (ISO 6888-1, 1999) [34]. Procedures to detect anaerobic sulfite-reducing bacteria (ISO 15213, 2003) [33] and *Bacillus cereus* (ISO 7932, 2005) [35] were applied. For a challenge test, enumeration of *Listeria innocua* and *Salmonella* spp. viable cells were performed by serial dilution and direct surface plating in duplicate onto Agar Listeria Ottaviani & Agosti (Biolife, Milan, Italy), according to ISO 11290-2 [36], and onto XLD agar (Oxoid, Italy). After counts, log CFU/g were calculated for replicates.

To evaluate the natural contamination of meat, analyses were performed in 25 g of sample for *Listeria monocytogenes* (ISO 11290-1, 2017) [37] and *Salmonella* spp. (ISO 6579-1, 2017) [38].

The pH values were obtained by directly inserting the tip of the electrode pH 127-m (692 pH/Ion Meter-Metrohm, Laramie, Wyoming, USA) into different portions of the samples. For challenge test, the pH was measured on 10 g of each sample using an HI 223 Calibration Check™ Microprocessor pH meter (Hanna Instrument, Smithfield, RI, USA) equipped with a Gel-Glass electrode (Hamilton, Bonaduz, Switzerland).

Water activity was measured at 25 °C by means of the a_w_-meter AQUALAB Series 3 Model TE (Decagon Devices, Inc., Pullman, WA, USA), according to ISO procedures (ISO 18787, 2017) [39].

Means and SDs were calculated, as were both an ANOVA test (*p* < 0.05) and Tukey’s test, with the use of InfoStat Statistical Software (Universidad de Cordoba, Cordoba, Argentina).

Normality of pH and a_w_ and log transformed microbiological data were assumed, due to the continuum scale of each variable and normality distribution of mean values used for comparisons (central limit theorem). In any case, in each one way ANOVA comparison, the Bartlett’s test for equal variances was tested (*p* < 0.05) and when homocedasticity was not observed a not parametric Kruskal-Wallis test (*p* < 0.05) was used by means of Intercooled STATA 7.0 (Statacorp).

### 2.3. Sensory Study

A panel of eight trained judges evaluated fermented sausages after 35 days of ripening by a preference analysis. Thus, the ten batches (1, 2, 3, 4, and 5 from both C and T groups) were analysed considering the liking of taste (sweetness, sourness, saltiness and spiciness), appearance, aroma, and overall acceptance. These attributes were rated from 1 (lowest qualification) to 9 (maximal qualification) in a hedonic scale. The sensory panel received slices of approximately 5 g of fermented sausages, without casings, in a sensory room with appropriate light. Samples were served at room temperature on white plastic dishes, coded with a three-digit number. Data were collected, means and SDs were calculated, as were both an ANOVA test (*p* < 0.05) and Tukey’s test using InfoStat Statistical Software (Universidad de Cordoba, Argentina). From the raw data, a Principal Component Analysis (PCA) was performed also using InfoStat Statistical Software. Normality of sensory dataset was tested by Shapiro-Wilk test, null hypothesis states that the variable is normally distributed with *p* < 0.05.

### 2.4. Challenge Test

#### 2.4.1. Preparation of Inoculums

The complete list of the strains used for the inoculations of *Listeria innocua* and *Salmonella* spp. is reported in Table 1. A mixture of three strains of *Salmonella*: *S. enterica* serovar Derby strain 106463/1 and monophasic *S. enterica* serovar Typhimurium antigenic formula 1,4, [5], 12:i: 118174/1 belonging to the IZSLER collection (Istituto Zooprofilattico Sperimentale della Lombardia ed Emilia Romagna, Italy) isolated from pork meat and fresh pork sausage, respectively and *S. enterica* serovar Typhimurium ATCC14018, were used. For *L. innocua* inoculation, five strains IZSLER 111373/1, IZSLER 111373/2, IZSLER 257529/1 and IZSLER 257529/2 (isolated from pork meat), and ATCC 33090 were employed as the surrogates of *L. monocytogenes*.

Strains were individually inoculated in Brain Heart Infusion broth (BHI, Oxoid) and incubated at 30 °C for 24 h in aerobic conditions. The bacterial cultures were prepared as reported by Bonilauri et al. [24] to obtain for each strain a concentration of about 10^9^ CFU/mL. Then, each strain was mixed together in order to achieve a final concentration of approximately 10^7^ CFU/g of each cocktail in the sausage mixture.

#### 2.4.2. Production of Nitrite-Free Fermented Sausages and HPP Treatment

A meat batter of 60 kg (*n* = 3) was prepared according to the basic recipe described below, with the addition of lactic acid 3%. The batter was thoroughly mixed and then divided into two batches: one inoculated with the mix of *L. innocua* (L) and the other inoculated with the mix of *S. enterica* (S). Before inoculation, five samples (around 25 g) of each batch were investigated for the presence/absence of *Listeria* sp. (ISO 11290-1, 2017) [37] and *Salmonella* spp. (ISO 6579-1, 2017) [38] to evaluate the natural contamination of meat.

The cold ripening process was applied to all sausage samples: 6–8 °C and 65–90% RH for 20–25 days, and 10–12 °C and 75–85% RH for 10–12 days. After 35 days, samples were peeled and vacuum packed (GK600/610 B Series, SUPERVAC, Mödling, Austria), and half of the samples of each batch S and L were stored at 12–14 °C for other 30 days (t_65_), whilst the other half were submitted to HPP treatment (Iperbaric, Burgos, Spain) using 593 MPa for 290 s and water at 14 °C.

Samples were analysed in triplicate at time 0 (before stuffing), 6 days (at the end of acidification step), 35 days (before and after HPP treatment), and 65 days for the non-treated samples with HPP for microbiological and physicochemical studies. Then, we determined the bacteria variations due to ripening, HPP, or storage by calculating the difference (Δ) of the average counts for two different samplings (N_1_, N_2_) expressed in log CFU/g, as follows: Δ = log (N_2_/N_1_).

## 3. Results and Discussion

**Figure 1:**
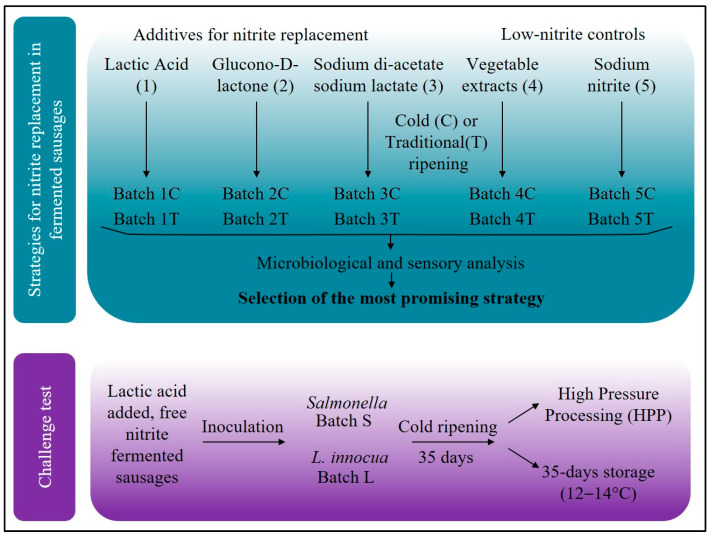
Workflow of the experimental design showing the two steps of this study: at first a comparison among additives and ripening conditions to select the most promising nitrite-free fermented sausages and at the second step it was submitted to challenge test. Additives used for different batches are indicated as 1: lactic acid; 2: glucono-D-lactone; 3: sodium acetate/sodium lactate; 4: nitrites from vegetables (80 ppm); 5: sodium nitrite (80 ppm). Groups following different ripening conditions are indicated as T: traditional ripening; C: cold ripening. Batch S: inoculated with *Salmonella* spp. Batch L: inoculated with *L. innocua*.

The complete workflow of this study is represented in Figure 1.

### 3.1. Effect of Additives and Ripening Conditions on Fermented Sausages Quality

#### 3.1.1. Microbiological and Physicochemical Analysis

Enumeration of main bacterial groups of fermented sausages was performed for samples containing different additives in alternative to nitrite: lactic acid, glucono-D-lactone, and a mix of sodium di-acetate and sodium lactate. In addition, two different low-nitrite fermented sausages were used as controls, both containing 80 ppm of nitrite, either as natural extracts or as inorganic nitrite (NaNO_2_). Each one of these five different batches followed a cold (C group) or a traditional (T group) ripening. Results for the ten batches along the 35-day ripening conditions are shown in Table S1. Counts of lactobacilli for the ten batches started with values in the range of 6.51 ± 0.01 log CFU/g to 6.62 ± 0.06 log CFU/g, without significant differences among samples. With regard to the C group, no significant differences were observed among samples with or without nitrites after 30 days, reaching counts between 8.07 ± 0.16 log CFU/g and 8.83 ± 0.62 log CFU/g. In addition, these values were similar to those found for the T group at 30 days. However, growth trends exhibited certain differences between samples with the same additive but with diverse ripening. In fact, the highest number of lactobacilli was achieved at day 30 for the C group, while it was registered earlier (at t_15_) for the T group, as expected. CNS counts at the initial time were in a range of 6.31 ± 0.14 log CFU/g and 6.52 ± 0.07 log CFU/g for the ten batches, without significant differences among them. In general, counts increased until day 4, and then remained almost unchanged for all batches. However, as an exception, the low-nitrite samples from the C group (batches 4C and 5C) presented the maximum values after 15 days. CNS counts presented values comprised between 7.15 ± 0.68 log CFU/g and 7.89 ± 0.03 log CFU/g at the end of the ripening for all the batches. No significant differences among them were observed, neither for nitrite-free sausages with the same additives but different ripening conditions. Interestingly, even though temperature is a main factor in the regulation of bacteria growth, the starter cultures seemed to be appropriately adapted to the cold ripening, as registered in our previous work [30]. In addition, our results showed the lack of nitrite influence on lactobacilli development; still, some effect could be noticed for staphylococci in samples following the cold ripening, since a slower growth was observed for batches with nitrites compared to nitrite-free sausages. Controversial results about the influence of nitrite/nitrate in staphylococci development have been found, as already discussed in Christieans et al. [5]. In this sense, Hospital et al. [6] reported staphylococci inhibition at the end of ripening using higher concentrations of nitrite and nitrate (150 ppm each) than those used herein.

For *Enterobacteriaceae*, counts were lower than or equal to 100 CFU/g for all the batches at the initial time, demonstrating a remarkably high quality of raw materials, and particularly, of the pork meat used. Counts < 10 CFU/g were obtained at 15 and 30 days for the T and C groups, respectively. *Enterobacteriaceae* inhibition coincided with the maximal growth of lactobacilli that usually exerts some antimicrobial activity due to the acidification of the meat matrix and microbial competition. It could be underlined that no differences were found among nitrite-free and low-nitrite samples at the same ripening condition. Therefore, low temperatures, not nitrites, mainly affected *Enterobacteriaceae* viability in this study, where high quality raw materials were used.

A similar drop was obtained only when high amounts of nitrate/nitrite (150 ppm/125 ppm) were used in Fabriano-like fermented sausages at the same time of ripening [40]. These results allowed us to suggest that temperatures and raw materials quality seemed to be the key variables that contributed to safety enhancement of nitrite-free sausages. Besides, *E. coli*, as well as vegetative cells and spores of both sulfite-reducing bacteria and *B. cereus*, were always lower than 10 CFU/g, whereas *L. monocytogenes* and *Salmonella* spp. were not detected in all samples during the whole process. Therefore, no food safety issues could be attributed to these fermented sausages, even in the absence of nitrites.

Regarding physicochemical parameters (Table S2), samples showed values in the range of 5.14 ± 0.03–5.69 ± 0.01 for pH and 0.975 ± 0.001–0.980 ± 0.002 for a_w_ at the initial time. It is worth noting that the addition of lactic acid and glucono-D-lactone (batches 1 and 2) led to pH values ≤ 5.2 at the beginning of fermentation, and then no further pH reductions were observed along ripening in both conditions. This acidification was conducted to inhibit pathogen bacteria from forming. In fact, Mataragas et al. [41] highlighted the importance of rapidly decreasing the pH in the first 48 h of fermentation for a quicker inactivation of *L. monocytogenes*, as had occurred in *salame Cacciatore*. In this work, the pH values of batches 3, 4, and 5 (those not having acidifying additives) were closely related to temperature and lactobacilli counts, instead. The physicochemical parameters of cold ripened samples exhibited slightly higher pH and a_w_ values than those traditional ripened samples at t_15_ and t_30_, reaching final values between 5.33 ± 0.04 and 5.48 ± 0.01 for pH and between 0.894 ± 0.003 and 0.898 ± 0.001 for a_w_. Results obtained for the cold ripening can be considered satisfactory for fermented sausages and appropriate to prevent undesired bacteria growth when high quality raw materials are used.

#### 3.1.2. Sensory Study

**Figure 2:**
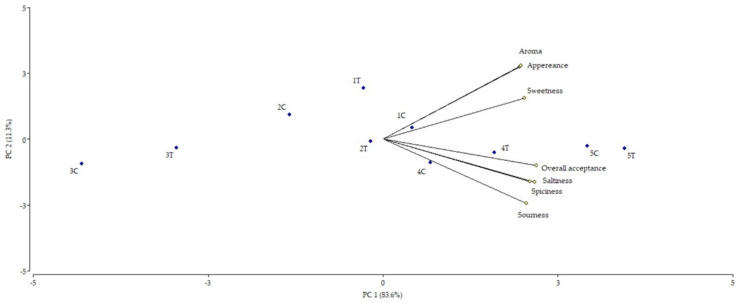
Principal component Analysis (PCA) of the sensory study conducted to the ten batches considering appearance, aroma, sweetness, spiciness, saltiness, sourness, and overall acceptance.

A trained panel performed a preference study of samples from the ten batches at the end of the ripening. The analysed attributes included sweetness, sourness, saltiness, spiciness, appearance, aroma, and overall acceptance (Figure S1). These attributes were considered to find the most preferred cold ripened nitrite-free sausages in terms of organoleptic characteristics. The whole data were analysed by using Principal Components Analysis (PCA) and graphically represented by a biplot (Figure 2) that accounted for a total of 95% of the explained variability. This PCA showed that the panel most preferred the two samples containing inorganic nitrites (batches 5C and 5T), followed by the samples with natural nitrites (batches 4C and 4T). For nitrite-free sausages, samples containing lactic acid from the C group (batch 1C) followed by the glucono-D-lactone-added sample from the T group (batch 2T) were the best scored. On the contrary, the samples containing sodium di-acetate and sodium lactate from both groups (3C and 3T) were the worst rated, suggesting that these compounds might affect sensory traits.

From the evaluated attributes, appearance that includes diverse characteristics, such as colour, cohesiveness, and oily appearance, has an especial interest. Nitrite is a key component for colour development of fermented sausages due to the nitrosomyoglobin pigment, which gives sausages their typically cured appearance. Nitrite elimination often leads to colour depletion, and its replacement, often with natural antimicrobials, rarely contributes to colour formation [8,42,43]. Many efforts have been conducted to evaluate natural substitutes for nitrite in diverse meat products that could provide other additional benefits, but colour remains an issue [44,45,46]. Considering appearance in our study, the best scored nitrite-free sausages were those with lactic acid: 8.00 ± 0.00 for batch 1C and 7.80 ± 0.45 for batch 1T. These values were similar to samples with inorganic nitrite (8.20 ± 0.45 for 5C and 8.00 ± 0.71 for 5T) and even better than those with added natural nitrites (6.80 ± 0.84 for batch 4C and 7.40 ± 0.55 for batch 4T). In addition, values for samples with lactic acid were better scored than glucono-D-lactone (7.20 ± 0.44 for batch 2C and 6.60 ± 0.45 for batch 2T) and sodium di-acetate and sodium lactate (4.80 ± 0.44 for batch 3C and 5.40 ± 0.54 for batch 3T). It is worth noting that lactic acid likely improved the sausages appearance, without drawbacks in taste, as not clear differences in sourness were found among samples. From these results, lactic acid represents a suitable alternative to nitrite-free fermented sausages. Furthermore, the combination of these two hurdle technologies (i.e., lactic acid and cold ripening) might be a promising strategy for the production of nitrite-free sausages based on microbiological, physicochemical, and sensory traits, including low hardening and oxidation, as previously determined by metabolomics and T-BARS studies [30].

### 3.2. Effect of Ripening, HPP, and Storage on L. innocua and Salmonella spp. Growth in Nitrite-Free Fermented Sausages

Challenge tests against *L. innocua* sp. and *Salmonella* spp. were applied to nitrite-free fermented sausages following our previous work [30]. Before inoculation, the meat batter was investigated for *Listeria* sp. and *Salmonella* spp., and their absences in 25 g of sample were verified. For each challenge test, a cocktail of strains was prepared and then inoculated (around 7 log CFU/g), obtaining two batches, L and S, for *L. innocua* and *Salmonella* spp., respectively. Samples were subjected to 35 days of cold ripening, and then the HPP treatment (593 MPa for 290 s) to the half of them was applied. The non-treated samples were stored at 12–14 °C in vacuum packaging for an additional 30 days. Counts of *Listeria* sp. or *Salmonella* spp., lactobacilli, and staphylococci, as well as pH and a_w_ values, were assessed after 0, 6 days, 35 days (before and after HPP treatment), and 65 days (for the non-treated samples) (Table 2).

Considering media values of both L and S batches, lactobacilli reported values of 6.96 ± 0.09 log CFU/g at the initial time and achieved 8.00 ± 0.29 log CFU/g at the end of ripening (Table 2). After the pressurisation, lactobacilli suffered a decline of 1.27 log CFU/g, while their counts were quite stable (reduction of 0.54 log CFU/g) after storage for non-treated samples. CNS started with values of 6.62 ± 0.10 log CFU/g and increased until 7.27 ± 0.35 log CFU/g at the end of ripening. Then, HPP led to a decrease of 3.54 log CFU/g. Conversely, a reduction of 1.87 log CFU/g at the end of storage for non-treated samples was observed. The significant effect of HPP on lactobacilli and staphylococci allowed us to infer that this treatment could be used only when these technological microorganisms have already accomplished their function, as Marcos et al. [47] had previously observed. Regarding physicochemical parameters, media values for both L and S batches started at 5.22 and 0.971 for pH and a_w_, respectively. While minor changes in pH values were registered (minimum pH value of 5.12 ± 0.02 was achieved at the 20th day, data not shown), the a_w_ values decreased until 0.896 at the end of the ripening. These parameters remained almost invariable in the vacuum package during storage at low temperatures. In general, pH and a_w_ values followed similar trends to those found in the previous trials described herein.

For the batch L, *L*. *innocua* strains were inoculated in a media concentration of 7.12 log CFU/g and correctly homogenised as SD was <0.5 during the whole experiment (Table 2). A reduction of *L. innocua* counts of 0.71 and 1.58 log CFU/g after fermentation phase (t_6_) and at the end of ripening, respectively was observed. When sausages were subjected to HPP (at pH 5.24 and a_w_ 0.895), an additional inactivation by 3.16 log CFU/g was obtained, achieving a total reduction of 4.74 log CFU/g for the whole process. On the other hand, *L. innocua* counts also exhibited a significant decrease (0.76 log CFU/g) during storage (non HPP-treated samples).

The cold ripening process induced a higher inactivation of *L. innocua* (Δ ripening = 1.58 log CFU/g) compared with other author findings for products with similar characteristics. For instance, Bonilauri et al. [25] reported an inactivation less than 1 log CFU/g of *L. innocua* during ripening for three diverse samples of *salame Cacciatore* with similar calibre (38 mm), ripening time (34 days), and final a_w_ value (0.896). These lower values could be related to the higher pH values during fermentation (5.38) and seasoning (5.52). In addition, 1 log CFU/g was the maximal reduction of *L. monocytogenes* for French products after 35 days of ripening but only using of 80/80 or 120/120 ppm of NaNO_3_/NaNO_2_ [5]. Our results for *L. innocua* strains are similar to those reported for *L. monocytogenes* in fermented sausages [5,48].

All these facts highlight the main importance of the EC Regulation 2073/2005 [49] that established the no-growth limits for *L. monocytogenes*: pH ≤ 4.4 or a_w_ ≤ 0.92 or pH ≤ 5.0 and a_w_ ≤ 0.94. Regarding HPP, our treatment greatly improved the rate of *L. innocua* inactivation (Δ HPP = 3.16 log CFU/g) in nitrite-free fermented sausages, whereas the complete inactivation (Δ ripening + HPP = 4.74 log CFU/g) allowed us to achieve decay values near the exportation requirements for the U.S. (reduction ≥ 5 log). On the other hand, Bonilauri et al. [25] observed a clearly minor inhibition of *L. innocua* (1.60 log CFU/g) after HPP treatment (600 MPa for 300 s) in *salame Cacciatore*. Besides, controversial results could be found in literature about the inhibition of *L*. *monocytogenes* in other meat products by HPP. For instance, high levels of inactivation (around 4 log CFU/g) were reported in RTE meats using 600 MPa, 180 s, and 20 °C [50], while no inactivation was detected in sliced fermented sausages (600 MPa, 5 min, 12 °C), which was attributed to low a_w_ (around 0.880) values and lactate content [26]. In general, these differences could be due to diverse a_w_ values_,_ solute composition, and pH parameters, as well as strain-dependent resistance to HPP. Moreover, even when a notable reduction by HPP was obtained in our study, the inhibition throughout under-vacuum storage (Δ storage = 0.76 log CFU/g) was not negligible. Recently, Gonzalez-Fandos et al. [51] reported a similar inactivation of *L. monocytogenes* in sliced Riojano Chorizo RTE fermented sausage stored for 28 days at 3 °C under-vacuum.

For the batch S, *Salmonella* spp., counts of 6.87 log CFU/g were found at the initial time, with an adequate SD for the entire experiment (SD < 0.5) (Table 2). After 35 days of ripening, a decrease of 0.85 log CFU/g was observed. A further 2.98 log CFU/g inactivation was obtained by HPP treatment when samples had a pH of 5.25 and a_w_ of 0.897. These two processes led to an overall drop of 3.83 log CFU/g for *Salmonella* spp. For the non-treated samples, the reduction of *Salmonella* spp. counts was 2.07 log CFU/g after the additional 30 days of storage. In general, the inactivation of *Salmonella* spp. might be enhanced by using high temperatures of fermentation due to a fast pH decline, as has often occurred in Northern European fermented meat products [52]. However, *Salmonella* spp. inhibition in Mediterranean sausages, where mild temperatures are regularly used, has been mostly attributed to a_w_ value diminution, but many factors are implied [53]. For instance, *S*. *Typhimurium* experienced a comparable decreased inhibition rate in French fermented sausages after 35 days of ripening to the effect of 80/80 ppm of NaNO_3_/NaNO_2_ (0.86 log CFU/g) or 120/120 ppm of NaNO_3_/NaNO_2_ (1.03 log CFU/g) [5]. When a large number of Italian sausages containing nitrite/nitrate was studied, *Salmonella* spp. reduction was in the range of 0.70–3.32 log CFU/g, including a diminution of 1.40 log CFU/g in *salame Cacciatore* [24]. These authors found that inactivation had a high correlation with pH value at the end of acidification (4.78–5.39), a_w_ value at the end of ripening (0.881–0.949), seasoning duration (21–90 days), and salami calibre (60–120 mm).

Differences could be explained by the possible contribution of other factors beyond a_w_ value, such as starter cultures, antimicrobial products, or nitrite/nitrate effects, among others. Nevertheless, it could be underlined that a considerable inactivation level was achieved in our study after ripening (Δ ripening = 0.85 log CFU/g), which is especially impressive since no nitrates or nitrites were added. Regarding the impact of HPP on Batch S, this treatment provided a greater contribution to *Salmonella* spp. reduction (Δ HPP = 2.98 log CFU/g) than ripening or storage. Besides, this inhibitory effect was in the range reported by other authors for traditional Italian fermented sausages (5.84–1.87 log CFU/g), although a greater reduction (3.72 log CFU/g) in a similar sausage (*salame Cacciatore*) had been previously observed [24]. Interestingly, our study observed a notable decrease (Δ storage = 2.07 log CFU/g) of *Salmonella* spp. during storage that could be associated with the long exposure to low a_w_ values, considering that the non-growth-permitting a_w_ value is ≤0.94 for *Salmonella* spp. [54]. However, several studies have shown that reduced a_w_ values protect against the inactivation of *Salmonella* spp. in low-moisture foods (e.g., chocolate, nuts, etc.) [55,56].

Our findings are in line with those of Mataragas et al. [41], who observed that the a_w_ reduction had becomes more important than the reduction in pH value to control foodborne pathogens such as *Salmonella* spp. in fermented sausages slowly acidified with fermentation temperatures < 20 °C. Comparing *Salmonella* spp. and *L. innocua* inactivation, our results indicated clear differences in their trends during ripening and storage. *L. innocua* strains seemed to be more susceptible than *Salmonella* spp. in our particular ripening conditions (lactic acid addition, low temperatures, as well as slow decreases of pH and a_w_ values). This is in contrast with other author findings that suggested that Gram positive bacteria are more resistant than Gram negative bacteria to the hurdle technologies applied in standard conditions of fermentation and ripening [41]. On the contrary, *Salmonella* spp. seemed to be more susceptible than *L. innocua* during storage when the a_w_ value was lower, and the pH was slightly higher than ripening. These variations could be partially explained by differences among specie or strains of *Listeria* sp. and *Salmonella* spp., although the influence of the conditions applied in our study may not be discarded.

Considering HPP, there is much evidence that demonstrated the influence of several factors, such as sodium chloride, sugar, fat content, and low a_w_ value in treatment efficiency, against diverse microorganisms [57,58,59,60]. Our results exhibited divergences in the inactivation levels observed by diverse authors could be attributed to the particular characteristics of each product and its processing. It is worth noting that the concentration of the inoculum of *Salmonella* spp. and *L. innocua* used in these challenge tests was designed to explore the maximal inhibitory capability of HPP. The inactivation levels found in our work could be relevant against the actual contamination in the processing chain of fermented sausages. Therefore, we agree with the evaluation of HPP performance by challenge tests addressed to a particular product in the real conditions of consumption or commercialisation.

## 4. Conclusions

Our results confirmed that cold ripening, combined with the addition of lactic acid 3% in fermented sausages, might replace the effect of nitrite without affecting sausages appearance or microbiological and physicochemical traits. In addition, our study complements our previous findings where the metabolomic approach allowed a deep analysis of fermented sausages quality correlated to both correct microbial development and desired biochemistry reactions. Finally, our findings emphasised the value of HPP as a final hurdle technology in nitrite-free fermented sausages because it exerted a main role in *L. innocua* and *Salmonella* spp. control. However, the use of high-quality raw materials is still mandatory to assure safety of these products.

## Figures and Tables

**Table 1:** Strains used for inoculation of *L. innocua* and *Salmonella* spp.

Identification Number	Specie	Source of Isolation
IZSLER 111373/1	*L. innocua*	Environmental swab of sausage factory
IZSLER 111373/2	*L. innocua*	Environmental swab of sausage factory
IZSLER 257529/1	*L. innocua*	Fresh sausage
IZSLER 257529/2	*L. innocua*	Pork meat
ATCC 33090	*L. innocua*	Cow brain
IZSLER 118174/1	*S. enterica* subsp. *enterica* serovar Typhimurium	Fresh sausage
IZSLER 106463/1	*S. enterica* serovar Derby	Pork meat
ATCC 14028	*S. enterica* subsp. *enterica* serovar Typhimurium	Animal tissues

IZSLER: collection of Istituto Zooprofilattico Sperimentale della Lombardia ed Emilia-Romagna, Italy.

**Table 2:** Counts (log CFU/g) of *L. innocua*, *Salmonella* spp., lactobacilli, and staphylococci coagulase negative (CNS) and physico-chemical parameters during challenge test. Bacteria variations (Δ) [log (N_2_/N_1_)] were calculated by step.

**Time (Days)**	***L. innocua***	***Salmonella***	**Lactobacilli**	**CNS**	**pH**	**a_w_**
0	7.12 ± 0.01 ^e^	6.87 ± 0.05 ^d^	6.96 ± 0.09 ^a^	6.62 ± 0.10 ^c^	5.22 ± 0.01 ^a^	0.971 ± 0.001 ^c^
6	6.41 ± 0.03 ^d^	7.03 ± 0.08 ^d^	8.12 ± 0.24 ^c^	6.93 ± 0.18 ^c^	5.25 ± 0.02 ^a^	0.964 ± 0.003 ^c^
35 (before HPP)	5.54 ± 0.12 ^c^	6.02 ± 0.13 ^c^	8.00 ± 0.29 ^c^	7.27 ± 0.35 ^c^	5.25 ± 0.03 ^a^	0.896 ± 0.007 ^b^
35 (after HPP)	2.38 ± 0.42 ^a^	3.04 ± 0.07 ^a^	6.73 ± 0.42 ^a^	3.73 ± 0.54 ^a^	ND	ND
65 (Non-treated)	4.78 ± 0.17 ^b^	3.94 ± 0.26 ^b^	7.46 ± 0.24 ^b^	5.40 ± 0.71 ^b^	5.34 ± 0.03 ^b^	0.882 ± 0.008 ^a^
**Δ [log (N_2_/N_1_)]**	***L. innocua***	***Salmonella***	**Lactobacilli**	**CNS**		
Δ Ripening	−1.58	−0.85	1.05	0.65		
Δ HPP	−3.16	−2.98	−1.27	−3.54		
Δ Ripening + Δ HPP	−4.74	−3.83	−0.23	−2.89		
Δ Storage	−0.76	−2.07	−0.54	−1.87		
Δ Ripening + Δ Storage	−2.34	−2.92	0.51	−1.22		

Letters as superscripts indicate significant differences by column based on ANOVA and Tukey’s test (*p* < 0.05). HPP: High Pressure Processing. ND: not determined. Δ Ripening = log CFU/g at the time 0 − log CFU/g at the end of the ripening (35 days before HPP); Δ HPP = log CFU/g before HPP − log CFU/g after HPP; Δ Ripening + Δ HPP: sum of Δ Ripening and Δ HPP; Δ Storage = log CFU/g at the time 65 days − log CFU/g at the time 35 days (before HPP); Δ Ripening + Δ Storage: sum (log CFU/g) of Δ Ripening and Δ Storage.

## Data Availability

Not applicable.

## Supplementary Materials

The following are available online at https://www.mdpi.com/article/10.3390/foods10112617/s1, Figure S1: Spider chart of sensory attributes for the ten batches. Additives used for different batches 1: lactic acid; 2: glucono-D-lactone; 3: sodium acetate/sodium lactate; 4: nitrites from vegetables (80 ppm); 5: sodium nitrite (80 ppm). T: traditional ripening; C: cold ripening. Table S1: Counts (log CFU/g) of lactobacilli, staphylococci coagulase negative (CNS), and Enterobacteriaceae for the ten batches. Table S2: pH and aw values for the ten batches
