# Supplementary material for: Strategies for Nitrite Replacement in Fermented Sausages and Effect of High Pressure Processing against Salmonella spp. and Listeria innocua
Source: Foods. 2021 Oct 28;10(11):2617. doi: 10.3390/foods10112617 (PMC8617797; doi:10.3390/foods10112617)
Supplement: Supplementary file 1 [file foods-10-02617-s001.zip › Figure S1.pdf]

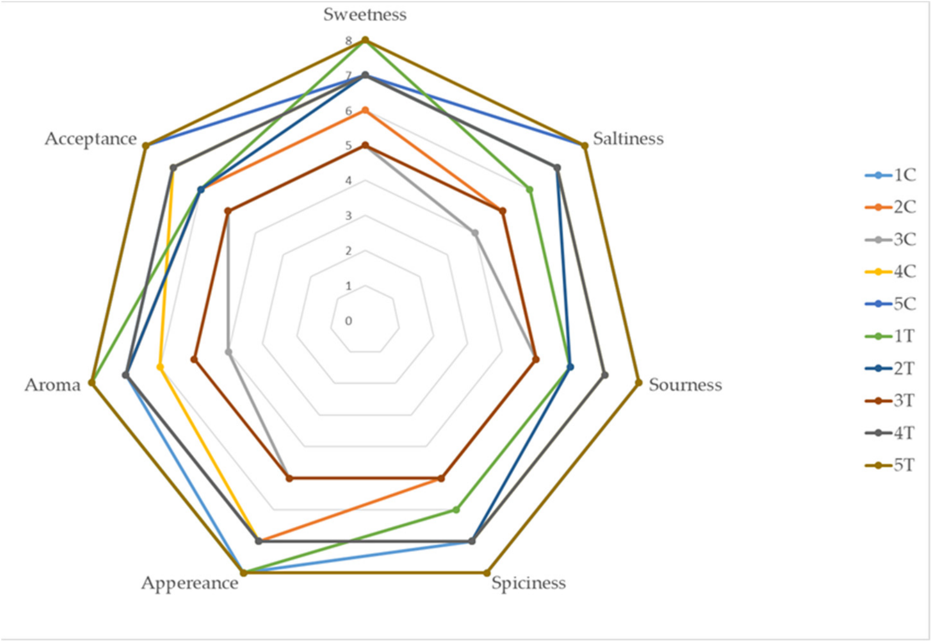

Figure S1: Spider chart of sensory attributes for the ten batches. Additives used for different batches 1: lactic acid; 2: glucono-D-lactone; 3: sodium acetate/sodium lactate; 4: nitrites from vegetables (80 ppm); 5: sodium nitrite (80 ppm). T: traditional ripening; C: cold ripening.
