# Supplementary material for: Strategies for Nitrite Replacement in Fermented Sausages and Effect of High Pressure Processing against Salmonella spp. and Listeria innocua
Source: Foods. 2021 Oct 28;10(11):2617. doi: 10.3390/foods10112617 (PMC8617797; doi:10.3390/foods10112617)
Supplement: Supplementary file 1 [file foods-10-02617-s001.zip › Table S1.pdf]

Table S1: Counts (log CFU/g) of lactobacilli, staphylococci coagulase negative (CNS), and *Enterobacteriaceae* for the ten batches.

Lactobacilli

Time (days)	1C	2C	3C	4C	5C	1T	2T	3T	4T	5T
0	6.57±0.04^{a,a}	6.51±0.01^{a,a}	6.54±0.06^{a,a}	6.62±0.06^{a,a}	7.07±0.73^{a,a}	6.57±0.04^{a,a}	6.51±0.01^{a,a}	6.54±0.06^{a,a}	6.62±0.06^{a,a}	7.07±0.73^{a,a}
4	7.84±0.08^{a,b}	7.87±0.01^{ab,b}	7.76±0.08^{a,b}	7.71±0.11^{a,b}	7.84±0.03^{a,a}	8.33±0.05^{c,b}	8.14±0.09^{bc,b}	8.36±0.05^{c,b}	7.89±0.05^{ab,b}	8.17±0.09^{c,a}
15	8.17±0.13^{abc,b}	8.24±0.06^{abcd,c}	7.98±0.02^{a,b}	7.92±0.08^{a,bc}	8.09±0.16^{ab,a}	8.62±0.08^{e,c}	8.47±0.06^{cde,c}	8.39±0.07^{bcde,b}	8.52±0.03^{de,c}	8.55±0.04^{de,b}
30	8.31±0.27^{a,b}	8.28±0.12^{a,c}	8.09±0.16^{a,b}	8.23±0.13^{a,c}	8.83±0.62^{a,a}	8.58±0.04^{a,c}	8.48±0.07^{a,c}	8.37±0.10^{a,b}	8.53±0.05^{a,c}	8.56±0.03^{a,b}

CNS

Time (days)	1C	2C	3C	4C	5C	1T	2T	3T	4T	5T
0	6.38±0.23^{a,a}	6.31±0.14^{a,a}	6.47±0.00^{a,a}	6.49±0.01^{a,a}	6.52±0.07^{a,a}	6.38±0.23^{a,a}	6.31±0.14^{a,a}	6.47±0.00^{a,a}	6.49±0.01^{a,a}	6.52±0.07^{a,a}
4	7.44±0.17^{abc,b}	7.48±0.09^{abc,b}	7.33±0.01^{a,a}	7.40±0.04^{ab,b}	7.45±0.10^{abc,b}	7.71±0.04^{cd,b}	7.69±0.03^{cd,b}	7.63±0.04^{bcd,b}	7.81±0.01^{d,b}	7.87±0.02^{d,b}
15	7.75±0.01^{ab,b}	7.73±0.02^{ab,b}	7.63±0.03^{a,a}	7.76±0.04^{ab,c}	7.78±0.03^{ab,c}	7.80±0.05^{ab,b}	7.88±0.04^{b,b}	7.78±0.10^{ab,b}	7.76±0.04^{ab,b}	7.83±0.06^{b,b}
30	7.61±0.02^{a,b}	7.66±0.06^{a,b}	7.37±0.68^{a,b}	7.53±0.04^{a,b}	7.58±0.10^{a,bc}	7.89±0.03^{a,b}	7.88±0.07^{a,b}	7.60±0.12^{a,b}	7.65±0.10^{a,b}	7.83±0.03^{a,bc}

Enterobacteriaceae

Time (days)	1C	2C	3C	4C	5C	1T	2T	3T	4T	5T
0	1.98±0.03^{a,b}	1.95±0.07^{a,c}	1.30±0.43^{a,b}	1.24±0.34^{a,a}	1.95±0.07^{a,c}	1.98±0.03^{a,b}	1.95±0.07^{a,c}	1.30±0.43^{a,b}	1.24±0.34^{a,a}	1.95±0.07^{a,b}
4	2.00±0.06^{a,b}	1.59±0.16^{a,b}	1.70±0.00^{a,b}	0.74±1.04^{a,a}	1.65±0.07^{a,bc}	1.97±0.10^{a,b}	1.65±0.07^{a,b}	1.59±0.16^{a,b}	0.80±1.13^{a,a}	1.54±0.09^{a,b}
15	1.77±0.10^{d,b}	1.48±0.00^{c,b}	1.54±0.09^{c,b}	1.00±0.00^{b,a}	1.39±0.12^{c,b}	<1±0.00^{a,a}	<1±0.00^{a,a}	<1±0.00^{a,a}	<1±0.00^{a,a}	<1±0.00^{a,a}
30	<1±0.00^{a,a}	<1±0.00^{a,a}	<1±0.00^{a,a}	<1±0.00^{a,a}	<1±0.00^{a,a}	<1±0.00^{a,a}	<1±0.00^{a,a}	<1±0.00^{a,a}	<1±0.00^{a,a}	<1±0.00^{a,a}

Letters as superscripts indicate significant differences by column based on ANOVA and Tukey's test ($p < 0.05$) by row (first) and by column (second). Additives used for different batches are indicated as 1: lactic acid; 2: glucono-D-lactone; 3: sodium acetate/sodium lactate; 4: nitrites from vegetables (80 ppm); 5: sodium nitrite (80 ppm). Groups following different ripening conditions are indicated as T: traditional ripening; C: cold ripening.
