# Supplementary material for: Strategies for Nitrite Replacement in Fermented Sausages and Effect of High Pressure Processing against Salmonella spp. and Listeria innocua
Source: Foods. 2021 Oct 28;10(11):2617. doi: 10.3390/foods10112617 (PMC8617797; doi:10.3390/foods10112617)
Supplement: Supplementary file 1 [file foods-10-02617-s001.zip › Table S2.pdf]

Table S2: pH and a_w values for the ten batches

**pH**										
**Time (days)**	**1C**	**2C**	**3C**	**4C**	**5C**	**1T**	**2T**	**3T**	**4T**	**5T**
0	5.24±0.02^{a,a}	5.14±0.03^{a,a}	5.56±0.04^{b,b}	5.65±0.04^{bc,b}	5.69±0.01^{c,c}	5.25±0.04^{a,a}	5.14±0.03^{a,ab}	5.56±0.04^{b,c}	5.65±0.04^{bc,c}	5.68±0.04^{c,c}
4	5.24±0.02^{c,a}	5.13±0.03^{ab,a}	5.52±0.03^{d,ab}	5.60±0.02^{d,b}	5.56±0.03^{d,b}	5.17±0.03^{abc,a}	5.11±0.03^{a,a}	5.22±0.01^{bc,a}	5.19±0.01^{abc,a}	5.14±0.02^{ab,a}
15	5.19±0.01^{b,a}	5.19±0.01^{b,a}	5.42±0.02^{c,a}	5.36±0.03^{c,a}	5.34±0.03^{c,a}	5.16±0.01^{ab,a}	5.09±0.02^{a,a}	5.23±0.01^{b,a}	5.22±0.02^{b,ab}	5.17±0.01^{b,a}
30	5.35±0.01^{bc,b}	5.33±0.04^{b,b}	5.48±0.01^{d,ab}	5.47±0.02^{d,a}	5.43±0.02^{cd,a}	5.22±0.01^{a,a}	5.21±0.01^{a,b}	5.34±0.01^{bc,b}	5.33±0.04^{b,b}	5.30±0.02^{ab,b}

**a_w**										
**Time (days)**	**1C**	**2C**	**3C**	**4C**	**5C**	**1T**	**2T**	**3T**	**4T**	**5T**
0	0.977±0.001^{ab,d}	0.975±0.001^{a,c}	0.978±0.006^{ab,d}	0.978±0.001^{ab,d}	0.980±0.002^{b,d}	0.977±0.001^{ab,d}	0.975±0.001^{ab,d}	0.977±0.001^{a,a}	0.978±0.001^{ab,d}	0.980±0.002^{b,d}
4	0.965±0.005^{a,c}	0.962±0.004^{a,c}	0.960±0.002^{a,c}	0.959±0.003^{a,c}	0.954±0.004^{a,c}	0.965±0.002^{a,c}	0.965±0.001^{a,c}	0.511±0.633^{a,a}	0.961±0.003^{a,c}	0.965±0.001^{a,c}
15	0.936±0.001^{e,b}	0.935±0.002^{de,b}	0.936±0.003^{e,b}	0.928±0.001^{bc,b}	0.928±0.001^{cd,b}	0.929±0.001^{cd,b}	0.928±0.001^{cd,b}	0.930±0.002^{cde,a}	0.923±0.003^{a,b}	0.922±0.001^{ab,b}
30	0.898±0.002^{d,a}	0.894±0.003^{bcd,a}	0.897±0.001^{d,a}	0.897±0.002^{d,a}	0.895±0.004^{cd,a}	0.880±0.003^{a,a}	0.879±0.001^{a,a}	0.885±0.002^{abc,a}	0.882±0.005^{ab,a}	0.880±0.004^{a,a}

Letters as superscripts indicate significant differences by column based on ANOVA and Tukey's test ($p < 0.05$) by row (first) and by column (second). Additives used for different batches are indicated as 1: lactic acid; 2: glucono-D-lactone; 3: sodium acetate/sodium lactate; 4: nitrites from vegetables (80 ppm); 5: sodium nitrite (80 ppm). Groups following different ripening conditions are indicated as T: traditional ripening; C: cold ripening.
